# A Transformative Journey Through Support Group Participation: Narratives of the Wives of Individuals With Alcohol Use Disorder

**DOI:** 10.7759/cureus.57461

**Published:** 2024-04-02

**Authors:** Akhil P Joseph, Anithamol Babu, L T Om Prakash

**Affiliations:** 1 Department of Sociology and Social Work, CHRIST (Deemed to be University), Bengaluru, IND; 2 School of Social Work, Marian College Kuttikkanam Autonomous, Kuttikkanam, IND; 3 School of Social Work, Tata Institute of Social Sciences Guwahati Off-Campus, Jalukbari, IND

**Keywords:** artificial intelligence, narratives, problems, support group participation, alcohol use disorder, wives

## Abstract

The issue of alcohol use disorder (AUD) has received significant attention, with a primary focus on individuals directly afflicted by the disorder. This extensive focus, while necessary, often overlooks the profound impact that AUD has on the family unit, particularly on spouses who play a crucial role in the dynamics of coping and recovery. However, the psychosocial and emotional challenges encountered by wives of those with AUD have been largely neglected in both research and therapeutic interventions. This oversight not only minimizes their suffering but also overlooks their potential contribution to the recovery process, underscoring the need for a more inclusive approach to understanding and addressing AUD. Building upon this foundation, the current study delves into the less explored terrain of the psychosocial and emotional ramifications borne by wives of individuals suffering from AUD. By highlighting the pivotal role that these women fulfil in family dynamics, it seeks to shed light on the transformative effects of their engagement in support groups, aiming to demonstrate how these networks promote resilience, empowerment, and healing for both the women and their families, thus offering a more comprehensive perspective on AUD's impact on society. Employing a qualitative narrative research design, the study utilized purposive sampling to select 36 participants from the northern, southern, and central regions of Kerala, India. Data collection was conducted through in-depth interviews using a semi-structured interview guide. The interviews, conducted initially in the local language, were transcribed into English and analyzed using the constant comparative method, ensuring that ethical considerations were upheld throughout the research process. The results of the study illuminate the multifaceted challenges faced by wives of individuals with AUD, including financial burdens, domestic violence, marital discord, and psychosocial issues. Notably, the participants reported a significant positive shift in their lives following their involvement in support groups, experiencing enhanced mental peace and tranquillity. This transformation enabled some participants to resume their education, engage with the community as role models and leaders, and reconstruct their lives. Most participants viewed their support group participation as a pivotal moment of hope restoration in their lives. The study reveals the necessity for integrating culturally sensitive support mechanisms into rehabilitation programs for families affected by AUD, advocating for broader adoption of support groups that cater to the specific sociocultural dynamics of affected communities.

## Introduction

The debate over the health consequences of alcohol consumption continues, with public perceptions often skewed toward the social acceptability and purported health benefits of low to moderate alcohol intake, such as reduced risks of stroke, heart failure, type 2 diabetes, and overall mortality [1,2]. This perspective starkly contrasts with the severe implications of alcohol use disorder (AUD), which is identified as a significant global health burden, contributing to over 5% of the annual global morbidity and mortality [3]. The impact of AUD extends beyond the individual, inflicting profound psychosocial and emotional distress on family members, particularly wives [4,5]. These individuals face a gamut of adversities, including sexual assault, mistreatment, injuries, marital violence, psychological coercion, and aggression, which underscore the extensive psychological and social toll exacted by AUD [6,7]. Moreover, the societal stigma and isolation resulting from a partner's excessive alcohol consumption compound the emotional turmoil faced by these wives, leading to shame, embarrassment, and a sense of isolation [8].

In response to these challenges, support groups such as Al-Anon have been shown to significantly improve the well-being of wives of individuals with AUD in developed countries, aiding in the treatment process from initiation to management [9,10]. These groups facilitate a journey of self-recovery for the wives, promoting loving detachment, self-esteem, independence, and spiritual reliance while providing crucial information, practical assistance, and emotional support to manage daily traumas [11]. However, in developing countries like India, the focus on AUD treatments like medication, counselling, and community support programs, predominantly targets the afflicted individuals, with the psychosocial impacts on their wives receiving minimal attention [8]. This oversight highlights a significant gap in the support infrastructure, underscoring the need for a more inclusive approach to AUD treatment that addresses the needs of both individuals with AUD and their wives. Thus, this study aims to shed light on the experiences of wives of individuals with AUD participating in support groups, advocating for the critical role that these groups play in providing a comprehensive support system that addresses the multidimensional challenges faced by these women.

## Materials and methods

Research design

The current study followed a narrative research design over other qualitative research designs to understand the problems faced by wives of individuals with AUD and the transformation of their lives through support group participation. The researchers considered narrative research design since it is extensively used to understand, describe, and examine the psychosocial problems and personality changes due to psychosocial interventions among men, women, and children [12,13].

Recruitment of participants

The current study followed a purposive sampling technique for selecting 36 participants, which exceeded the minimum sample size of 20 as Creswell recommended for conducting semi-structured interviews [14]. Kerala is divided into the north, south, and central regions for choosing participants. Four support groups were selected from each region, linked with the Integrated Rehabilitation Centre for Addicts (IRCA), Kerala, India. The participants were taken from each region with the help of four IRCA heads with 15 years of experience, assuming that support group processes are effective in such groups with a long existence. The researchers selected three participants from each support group as recommended by the facilitators of the support groups satisfying the following criteria: a) age group of 30-60 years; b) having at least two children and a husband, who was alcohol-dependent; c) minimum of two years of participation in a support group, and d) should reside in Kerala, India. Participants with indications of severe physical and mental illness, including mood disorders and cancer, were excluded from the study. Thus, 12 participants in each region were selected through expert analysis by considering facilitators as experts based on the inclusion criteria.

Tool

The researchers employed an in-depth interview method for data collection, utilizing a semi-structured guide that included demographic questions and open-ended inquiries into the participants' psychosocial problems, motivations for joining the support group, and life experiences following their involvement. The interview format was framed in such a way that the participants were encouraged to share more information with probes like “Could you tell me more about that?” and “Could you give me an example?”, which were also used to help each participant to continue and illustrate their narratives.

Data analysis

The data were analyzed using a constant comparative method associated with grounded theory, which used outside grounded theory as a qualitative analysis method to accept social reality as a logic of discovery [15]. Therefore, to explore, elaborate, and systematically process the data with the similarities and dissimilarities, the researchers transcribed one interview from the local language (Malayalam) to English. The interview transcript was analyzed to generate codes that uncovered the experiences of the wives of individuals with AUD. The data from the remaining interviews of the participants were compared with the codes generated from the first analyzed interview, and the embedded codes were identified. The codes were then grouped into themes, and patterns were identified to explain the experiences of the wives of individuals with AUD.

Ethical considerations

The researchers received ethical approval from Christ College and Christ University (CHRIST) (Deemed to be University), Bengaluru, India (CU: RCEC/00333/06/22). Voluntary consent was obtained from the participants by agreeing on the conditions that the participant voluntarily agrees to participate in the research, does not benefit from the research, and has the right to withdraw during the study. The voice recordings of the interviews, with participant consent, were saved on Google Drive using a Gmail account created exclusively for research purposes.

## Results

Characteristics of the participants

Among the 36 participants, half (18) were in the age group of 30-40 years, while the remaining half (18) were in the 40-60 age group. Twenty participants were Christians, 11 were Hindus, and five were Muslims. Except for one participant, all the others had marriages arranged by their family. Currently, three-quarters of the participants (27) live in nuclear families, and a quarter (nine) live with extended families. All the participants passed matriculation; among them, 16 had qualified for their higher secondary examination, and nine had graduated. Concerning occupation, nearly three-quarters (26) of the participants were housewives, two were entrepreneurs, three were government employees, and the remaining five worked as salesgirls in shops. More than one-third (14) of the participants had three children, seven had four children, and the remaining had two children each. Fifteen participants have participated in the support group for the last two years; another 15 have participated for a duration ranging from two to four years, while six have participated for more than four years.

Discovery of husband’s alcohol dependence

All participants revealed that their husbands had been dependent on alcohol since adolescence. A significant majority (27 out of 36) reported that before their marriage, when their families inquired about the groom and his background, he was often described as "not a drinker… but just an occasional social drinker." Only one participant, who married for love, was aware of her spouse's AUD from the beginning of their relationship. The others were unaware of their future husbands' alcohol dependence until after marriage. Two participants mentioned that their husbands had sought treatment in de-addiction centers before their marriage, with one recounting how her "honeymoon" was spent visiting various de-addiction facilities across Kerala for her husband's treatment.

Problems faced by the wives before participating in the support groups

The study found that all participants had suffered emotional and verbal abuse from their husbands, with over two-thirds (29) experiencing physical abuse. All participants, except one, reported that their husbands had forcefully taken their earnings or gold ornaments to purchase alcohol. Seven participants faced the harrowing situation of their husbands' creditors demanding sexual favours as repayment, shockingly, sometimes in front of their semi-conscious husbands. They expressed their disbelief and moral outrage, questioning, "How could a woman bear to hear something as immoral as this in front of their husband and children?" Also, nine participants felt humiliated by becoming a "laughingstock" at social events due to their husbands' absent-mindedness (indicative of memory impairment). A third of the participants (12) noted that their husbands' tendency to start quarrels at social gatherings led them to "stop attending functions of friends and relatives" altogether to avoid further embarrassment. A particularly disheartening revelation from the participants was the blame they shouldered for their husband's alcohol dependence. Despite being uninvolved in their husbands' decisions to drink, the women endured the burden of societal judgment. One participant lamented, "Despite my hardships, society blames me for his addiction. Even though his habits were known before our marriage, I'm seen as at fault, as if I'm the one providing his drinks."

Reasons for not leaving an alcohol-dependent husband

All participants contemplated leaving their husbands, pushed to their limits by their suffering, and some considered suicide to escape their agony. However, the concern for their children's welfare - who would look after them - prevented them from taking such extreme measures. Sharing her agonizing dilemma, one participant disclosed, “Many nights, the thought of ending it all seemed like the only way out. But the image of my children's faces would flash before me. ‘Who will look after my children if I commit suicide?’ That haunting question became my lifesaver. They needed me.” Despite their challenges, many participants believed that their love and care might eventually lead their husbands to change. Their optimism stemmed partly from a desire to preserve the family unit for their children. One participant said, “During our darkest moments, I gathered my children and asked them, 'If your father and I were to separate, who would you want to stay with?’ Their teary eyes, looking at me, were my answer. I realized I needed to continue enduring pain for them.”

In their quest for a solution, more than half of the participants (20) explored a variety of treatments to combat their husbands' AUD, ranging from traditional Ayurveda and homeopathy to conventional allopathy. Moreover, two-thirds of the participants sought solace and potential remedies in religious practices, turning to rituals and prayers from Hinduism, Christianity, and Islam. Their faith was often bolstered by testimonies from others who had witnessed positive changes, prompting them to cling to the belief that spiritual intervention could make a difference.

Experiences in the support groups

All participants attributed their decision to join the support group to the influence of senior members, especially during times of crisis. One participant vividly recalled, "I thought if it helps me, I could try this too as a last-ditch attempt… and that was the best decision I had made in my life." This sentiment underscores the pivotal role of support and guidance from more experienced members, who provided a non-judgmental and accepting environment in the support group. The sharing of "life experiences" by these senior members, coupled with the "constant support" from the group, emerged as a beacon of hope for the newcomers, significantly impacting their lives from the moment they began participating in support group meetings. One participant commented, "The group made me see that I am more than just a victim. I have strength and resilience." She highlighted the pivotal role of the group, stating that its members offer unwavering support, fostering a sense of empowerment among participants.

The transformative power of the support groups extended beyond the participants to influence community perceptions and interactions. One participant noted a dramatic shift in how their household was viewed, from being the "noisiest, chaotic household in the entire neighborhood" to the "most peaceful house in our locality," highlighting the cessation of domestic disturbances. The ceasing of arguments, as one participant shared, came from a newfound trust in their spouse, "I lived in constant fear he would return to drinking. Every time he left the house, I would argue with him. However, after joining the support group, I learned to trust him again, and the arguments stopped." This change led to a significant reduction in self-stigma and improved social interactions, with one-third (12) of the participants reporting that their neighbors had begun socializing with them, attracted by the newfound peace and calm in their homes. Two-thirds (22) of the participants observed a shift in their neighbors' perceptions, now seeing them as catalysts for their husbands' transformation, a stark contrast to the previous label of "chantha penungal" (loud and ill-behaving women) due to their confrontations with their alcohol-dependent husbands. More than half (21) of the participants experienced a substantial change in their "life situation and visibility" within their communities, marking a significant milestone in their journey toward recovery and social reintegration, facilitated by their active participation in the support group.

Participants in the study reported a profound sense of "mental peace and tranquillity" following their involvement in support groups, attributing this change to several key insights and transformations. First, they gained a sense of universalization, realizing that they were not alone in their struggles. This realization came from sharing experiences with others in similar situations, which provided a significant emotional relief. Many participants mistakenly believed that their husbands’ addiction was their fault or a sign of personal failure. However, through the insights from the support group, they realized that addiction, as a disease demanding multidimensional professional support, was another critical factor in their newfound peace. This shift in perspective allowed them to adopt self-care strategies, focusing on their well-being and their families for the future. Moreover, the support network facilitated personal growth opportunities, with three participants resuming their education and another starting to work and even purchasing a bike with the assistance of senior group members.

However, the experiences were not uniformly positive; two participants reported ongoing trauma, finding themselves unable to forgive or forget their spouses' past actions, even after the drinking had stopped. This underscores the complex emotional landscape these women manage, recognizing AUD as a disease with the potential for relapse. One particularly distressing account involved a participant whose husband, in a drunken state, physically harmed their 15-year-old child at night due to delusional jealousy (he thought she was having an affair with the child when she denied him sex). Her son continues to suffer due to the physical injury inflicted upon him. She voiced, "How will I ever forget what my husband had done to my child, even though he is a changed person now?." This incident left lasting physical and emotional scars, highlighting the severe impact of AUD beyond the individual sufferer to the family unit. These narratives underscore the critical role of support groups in providing a space for healing, understanding, and mutual support, yet also reveal the profound and sometimes irreversible damage inflicted by AUD on families.

## Discussion

The present investigation delves into the challenges faced by wives of individuals with AUD and the transformative impact of their participation in support groups. The institution of marriage in this region, predominantly arranged by the parents or guardians, involves meticulous background checks on the prospective spouses, a practice deeply rooted in social and familial norms [16,17]. Despite the social acceptability of male alcohol consumption as a marker of masculinity and social cohesion, families often conceal the drinking issues of bridegrooms to adhere to societal and religious matrimonial timelines [18]. This scenario underscores the societal pressure and the extent of emotional leverage parents exert over their children to fulfill their marital desires, often against the children's wishes, highlighting a pervasive issue of coerced marriages within the specified age bracket of 18-30 years, irrespective of the individuals' readiness or personal desires [19].

The study further illuminates the dire predicaments faced by women, where leaving a marriage marred by AUD becomes a last resort, overshadowed by the fear of societal stigma toward divorce and the repercussions of raising children in a single-parent household [20,21]. The societal stigma attached to being "divorced" and the subsequent vulnerabilities to sexual harassment exacerbate the women's plight, compelling them to endure the adversities within their marital confines [19]. However, participation in support groups leads to a significant transformation, including empowerment, enhanced self-esteem, and the development of practical strategies for personal growth. The support and shared experiences from senior members, who have previously confronted AUD, are crucial in driving the rehabilitation process forward [22]. This collective engagement not only facilitates a therapeutic catharsis but also fosters a sense of empathy, connectedness, and mutual support, thereby enhancing the understanding and relationships between the wives and their alcohol-dependent husbands [23].

The transformative journey facilitated by support groups in Kerala, when placed in a comparative global context, reveals a nuanced understanding of their efficacy and adaptability across diverse sociocultural landscapes. Studies in Western contexts emphasize personal empowerment and individual coping strategies, reflecting a cultural inclination toward individualism [9], whereas in collectivist societies like Kerala, the focus shifts toward communal healing and collective family well-being [24]. This comparative analysis underscores the universal benefits of support groups, namely, improved mental peace, familial relationships, and emotional well-being, highlighting the cultural specificity in the pathways to these outcomes. The variance in stigma associated with AUD and the participation in support groups across cultures impact engagement levels, with more conservative societies facing significant societal barriers to participation [25,26].

Furthermore, while the adaptability of support groups to cultural needs showcases their potential for empowering women in traditionally domestic roles across diverse settings, including Kerala, the study's qualitative methodology and its focus on a specific cultural context limit its generalizability [27]. This global perspective underlines the critical need for cultural sensitivity and adaptability in addressing AUD, suggesting that effective support mechanisms should not only be universal in their reach but also tailored to fit the diverse cultural contexts in which they operate. It advocates for a nuanced approach that respects and incorporates cultural values and norms, thereby enriching the international dialogue on best practices for support groups. Such an approach can significantly improve the efficacy of support mechanisms for families grappling with AUD by ensuring that interventions are culturally relevant and resonate more deeply with those they aim to help. The study underscores the critical role of support groups in addressing the co-dependency issues prevalent among the wives of individuals with AUD in the Indian context, advocating for the integration of such psychosocial interventions in rehabilitation centers and hospitals [28,29]. This necessitates a unified endeavor by mental health professionals to develop culturally attuned interventions, incorporating artificial intelligence (AI) to enhance the efficacy and accessibility of mental healthcare solutions [30]. Such integration will illuminate the path for extensive research on the enduring effects of support group engagement within institutional frameworks, significantly enriching the discourse on addiction, marital dynamics, and societal health.

## Conclusions

The study on wives of individuals with AUD in Kerala, India, reveals the critical role of support groups in navigating the challenges posed by AUD within marriages. These groups provide a platform for shared experiences, fostering resilience and empowerment among participants. The narrative underscores the importance of understanding sociocultural influences on marital dynamics and the effectiveness of communal support in facilitating recovery and empowerment. Highlighting the need for holistic support mechanisms, the study advocates for empathetic approaches in addressing the complexities of AUD in familial contexts.

Integrating AI into mental healthcare offers a promising avenue to enhance support for individuals and families affected by AUD. AI can provide personalized support, enable early intervention through predictive analytics, and improve access to mental health resources, complementing traditional support systems. By facilitating virtual support networks, AI can help overcome barriers related to stigma and accessibility, offering a scalable solution to meet the diverse needs of those dealing with AUD. This study paves the way for exploring AI's role in enhancing early intervention, improving access to mental health resources, and overcoming stigma-related barriers, aiming to advance mental health outcomes and social well-being by bridging technology with community-based support. Therefore, future longitudinal studies should explore the integration of AI to provide tailored interventions and access granular details, fostering deeper insights into the long-term impacts of support group participation and enhancing targeted support for AUD-affected families.
